# Factors for success in mental health advocacy

**DOI:** 10.3402/gha.v8.28791

**Published:** 2015-12-17

**Authors:** Katrina Hann, Heather Pearson, Doris Campbell, Daniel Sesay, Julian Eaton

**Affiliations:** 1 Mental Health Coalition – Sierra Leone, Freetown, Sierra Leone; 2 CBM International, Lomé, Togo; 3 Centre for Global Mental Health, LSHTM, London School of Hygiene and Tropical Medicine, London, UK

**Keywords:** global mental Health, mental health advocacy, mental health policy, self-advocacy, disability, community-based participatory research, Sierra Leone, Africa, LAMIC

## Abstract

**Background:**

Mental health advocacy groups are an effective way of pushing the mental health agenda and putting pressure on national governments to observe the right to health; however, there is limited research that highlights best practices for such groups in low-resource settings. In an effort to improve the scaling up of mental health in Sierra Leone, stakeholders came together to form the country's first mental health advocacy group: the Mental Health Coalition – Sierra Leone. Since its inception, the group has worked towards raising the profile of mental health in Sierra Leone and developing as an advocacy organisation.

**Design:**

The study's aim was to investigate views on enabling factors and barriers associated with mental health advocacy in a low-income country using a community-based participatory approach and qualitative methodology. Focus groups (*N*=9) were held with mental health stakeholders, and key informant interviews (*N*=15) were conducted with advocacy targets. Investigators analysed the data collaboratively using coding techniques informed by grounded theory.

**Results:**

Investigators reveal viewpoints on key factors in networking, interacting with government actors, and awareness raising that enabled mental health advocacy aims of supporting policy, service delivery, service user rights, training for service delivery, and awareness raising. The investigators outline viewpoints on barriers for advocacy aims in framing the issue of mental health, networking, interacting with government actors, resource mobilization, and awareness raising.

**Conclusions:**

The findings outline enabling factors, such as networking with key stakeholders, and barriers, such as lack of political will, for achieving mental health advocacy aims within a low-resource setting, Sierra Leone. Stakeholder coalitions can further key policy development aims that are essential to strengthen mental health systems in low-resource settings.

In Sierra Leone, mental health services are limited and outdated, despite the great need for mental health care. In 2002, the World Health Organization estimated that 500,000 people were affected by mental health problems; 2% of the population was suffering from psychosis, 4% severe depression, 4% substance misuse problems, 1% intellectual disability, and 1% epilepsy (1). The long-lasting violence of the 1991–2002 civil war left deep scars on the nation's psychological well-being. More recently, the country suffered a catastrophic Ebola virus disease outbreak, which has also had a profound impact on the well-being of individuals and communities (2). Existing services, limited to one psychiatric hospital, do not meet the needs either for specialist care or in terms of accessibility for the majority of the country. When measured using disability-adjusted life years, neuropsychiatric disorders represent the most disabling conditions among non-communicable diseases (3). From the economic and social perspective, there is strong evidence that this has a detrimental effect on a country's development and is a significant barrier to achievement of the global development objectives (3, 4).

Despite ratification of the Convention on the Rights of Persons with Disabilities (5) by Sierra Leone in 2010, persons with psychosocial disabilities are often ostracized from their communities, and human rights violations similar to those in other parts of the West African region, such as chaining or lack of access to evidence-based treatment, are common (6–10). Traditional beliefs and treatment approaches that attribute mental illness to spiritual causes and often blame the person living with the mental health problem contribute to these realities. Lack of public awareness and negative attitudes surrounding mental illnesses underlie high levels of stigma and discrimination against people with mental health problems in Sierra Leone (10).

## Mental health advocacy

Sierra Leone's challenges in mental health are not unique to the country. Globally, it is estimated that 30% of countries do not have mental health programmes, whereas 40% do not have mental health policies to inform service delivery. Within the African continent, care is primarily offered in psychiatric hospitals as more than 40% of countries have no community-based mental health services (11). The lack of prioritisation by government and key decision makers is identified as a significant barrier to scaling up mental health services (12). The empowerment of stakeholders as advocates is recognised not only as an effective tool to overcome this, but a fundamental principle (7).

Although this principle of ‘nothing about us without us’ is well recognised, people affected by services continue to have relatively little say in how those services are run. Stakeholders at all levels should have a central role in both advocating for reform and in participating in the processes of reform (13). Worldwide, it has become a consensus that mental health advocacy groups are an effective way of pushing the mental health agenda and putting pressure on national governments (14). User-led disability organisations and the self-advocacy movement have their origins in high-income countries. The evidence base of self-advocacy, mostly drawn from these areas (15–20), points to the effectiveness of groups of key stakeholders in pushing forward the mental health agenda (21).

However, research that highlights best practice for self-advocacy in low-resource settings is limited (22). Since 2010, a growing movement to establish and build capacity in mental health advocacy groups has championed the establishment of stakeholder advocacy groups across West Africa, including in Sierra Leone (23).

## Mental health advocacy in Sierra Leone

In response to the ongoing challenges in mental health in Sierra Leone, the Mental Health Coalition-Sierra Leone (MHC) (24) was founded in August 2011, with membership from service users and their family members, service providers, non-governmental organisations (NGOs), government officials, and civil society. The MHC's stated purpose is to create a national body that empowers stakeholders to advocate for their needs, thus raising the profile of mental health in Sierra Leone. Since its inception, the MHC actively developed as an advocacy movement with a constitution highlighting the organisation's goals (Table 1) (25). The group made significant efforts towards its advocacy aims, including promoting mental health in national-level policy initiatives, and, since the research study was undertaken, emerged as central to coordination of the mental health and psychosocial response to the Ebola crisis. The MHC convened the research study to link their perceived successful efforts and associated enabling factors and barriers, and to support the evidence base from low-resource settings on stakeholder advocacy for mental health.

**Table 1:** Goals of the Mental Health Coalition – Sierra Leone

Advocate with government bodies to pay more attention to mental health issues and work systematically to improve services for people with mental illness
Coordinate activities between NGOs and governmental agencies, allowing space for and facilitating networking
Empower stakeholders, particularly service users so that they can clearly voice their own priorities
Spread awareness about mental health and promote mental health in the general population
Support the empowerment of service users in Sierra Leone
Act as an advisory and monitoring body for the national mental health programme (strategic plan implementation, implementation of this project), and for other organisations requiring advice and information on mental health issues in Sierra Leone

## Methods

The study aims to identify views on factors associated with successful advocacy and related barriers using a community-based participatory research (CBPR) approach. Members of the MHC designed and carried out the study and were trained in research methods with a strong emphasis on capacity building in order to prepare community members for advocacy and community action that is based in evidence (26).

Investigators used Strauss and Corbin's grounded theory (27) in the context of a CBPR framework. The training of investigators, deliberately emphasised collecting data systematically and then using this data to develop a theory, rather than starting with a preconceived theory and testing with data. Research questions were developed collaboratively with the study team and other members of the MHC (Table 2). The research questions were then used to develop interview guides that focused on mental health advocacy in Sierra Leone, the role of the MHC, and enabling factors and barriers. In addition, the research team was encouraged to recognise that all data are relevant, and record observations as such, in keeping with grounded theory.

**Table 2:** Research questions and sample characteristics

Research questions	**RQ1** What, if any, are the advocacy successes of the MH Coalition since its inception? **RQ2** What, if any, are the factors associated with these successes? **RQ3** What, if any, are the challenges for successful advocacy by the MH Coalition since its inception? **RQ4** What, if any, are the factors associated with these challenges to advocacy success?	
Type of data collection	Sample	Number of participants
Key informant	Government representatives	6
interview participants	Tertiary education institutions	2
	Religious groups	2
	Non-governmental organisations	2
	Private sector service providers	1
	Development partners	1
	Traditional healer's associations	1
Focus group discussions	MHC members, female	4
	MHC members, male	7
	Ex-service users, female	1
	Ex-service users, male	3
	Family members of service users, female	8
	Family members of service users, male	6
	Service providers, female	8
	Service providers, male	6
	Freetown police, mixed-sex	8

The study team conducted key informant interviews (KIs, *N*=15) with MHC-identified key influencers to gain expert knowledge on potential advocacy impact; and focus group discussions (FGs, *N*=9) with key membership groups allowed for discussions to gain a diverse understanding from key membership and stakeholder groups. Potential KI participants were purposively sampled in collaboration with the MHC from a list of stakeholder contacts based on their area of influence – FG participants from lists of MHC members, stakeholder contacts based on the categories above and sex, as well as through snowball sampling. KIs and FGs lasted between 30 and 85 min and were conducted in the language of preference of the participant(s) (English, Krio, and/or Temne), in a private location accessible to the participant(s).

### Data collection and analysis

The findings of this study are based on qualitative data collection conducted between September and October 2013 in Freetown and Makeni, Sierra Leone. The team conducting data collection included a qualitative researcher, a public health specialist, and MHC members trained in qualitative research ethics and data collection over a period of 4 months. The study team debriefed daily during the period of data collection to reflect on progress, create memos on preliminary findings, and review possible saturation.

Recorded sessions were transcribed verbatim and translated into English by a native speaker of Krio or Temne, as required. Grounded theory guided the analysis approach, which was conducted using MAXQDA analysis software (28). The team reviewed all transcripts and debriefing notes without following any presumed theory, but rather looking at all existing data to identify initial units through open coding. In the open coding phase, coding notes or memos were developed, outlining the concepts underpinning each line or sentence in the transcript by research assistants with the support of the Principle Investigator. As this was done for all the data, general themes or categories that were noted to be similar or repeating emerged. Once all data had been coded in this way, the themes were re-examined in a process of axial coding, where the transcripts and memos were reviewed, and connections that explained the type of relationship between themes, were made. This process of comparison of raw data to emerging concepts was repeated several times until there was a good fit of data grouped into themes. One team member iteratively coded the data using the codes defined in the axial coding and completed selective coding based on emerging relationships between codes, which was reviewed by a second team member. The team utilized this approach to support the participatory approach to analysis. However, a limitation of the approach is that inter-rater reliability could not be established.

## Ethics

The Sierra Leone Scientific and Ethics Review Committee granted ethical approval for this research. The study team convened a Community Advisory Board consisting of representatives from the police force, service providing institutions, health advocacy groups, and service users and their families; and provided context-specific ethical guidance to the research team throughout the study.

Verbal informed consent was obtained from all participants, to allow for inclusion of participants with low literacy levels and to remove distrust associated with signing documents (29, 30), which included the permission to record the interviews. As a particularly vulnerable group, additional considerations were given to the inclusion of ex-service users. Based on the advice of the Community Advisory Board, the study relied on MHC community contacts to identify potential ex-service users who were no longer seeking care for inclusion in the study. A lunchtime meal and reimbursement of transport costs was provided for not only the participants themselves, but also for a caregiver, if the participants opted to have one accompany them to the location.

The study had the potential to reveal participants’ identities due to the small sample size and nature of many of the participants. To protect the confidentiality of the participants, the study team marked data with codes and replaced identifiable information with generic descriptors.

## Results

KI interviews (*N*=15, 13.3% female) and focus groups (*N*=9) with groups of up to 10 same-sex participants (with the exception of the Freetown Police, which was mixed sex at the recommendation of the Community Advisory Board) were completed (Table 2).

The results reflected the priorities and motivation of the participants, which grew out of the long CBPR processes of training and conceptualisation of the questions that underpinned the research. The research questions were generally focused on enabling factors and barriers (Tables 3 and 4) towards advocacy aims, as this is the recognised driver behind the organisation. Themes relevant to mental health policy and systems strengthening in Sierra Leone emerged, which is in line with the main work interviewed stakeholders have been engaged with. Out of the many specific examples of particular practice, some of which are given here, several overall themes emerged, focused on relevance and workability. The results did not lend themselves to an overarching theory that encompassed all findings, but instead a set of guiding principles. This was based on a clear desire for communication of experience to other comparable groups aiming to have an impact on policy and political will.

**Table 3:** Enabling factors for advocacy aims

Networking	Effectively coordinating common messages across a wide range of stakeholders, including: National stakeholders (Ministry of Health & Sanitation) First Lady of Sierra Leone Ministry of Social Welfare, Gender, & Children's Affairs International community Carter Centre (Liberia) Sierra Leonean diaspora West African links, West African mental health Leadership & Advocacy Programme (mhLAP)
Interaction with GoSL	Active involvement of the Ministry of Health & Sanitation ensured Government of Sierra Leone pushed little by little to acknowledge the issue
Awareness raising	Using opportunities to effectively communicate messages: Annual conference Communications: website, flyers, posters, newsletter MHC members as peer advocates Sensitization activities Workshops World Mental Health Day

**Table 4:** Barriers for advocacy aims

Framing the issue	Not a government or donor priority Gaining support for mental health, ‘getting everyone on board’ Identification of mental health as an issue
Networking	Not enough partners in mental health MHC activities not very well known about Traditional healers not included
Interaction with GoSL	Lack of planning for mental health Government required to take the lead Implementation of the Mental Health Policy Lack of political will or mindset change
Resource mobilization	Competition for resources in the health sector Lack of funds for mental health services Lack of resources for MHC advocacy activities
Awareness raising	Insufficient sensitization Lack of engagement with the media Need for a holistic view of mental health and services Stigma

A sex-specific analysis was conducted to understand if any themes emerged from participants of a specific sex, with no significant results.

### Advocacy aims

Participants referred to the MHC's identified achievements (Table 5). The launching of the Ministry of Health and Sanitation's (MoHS) Mental Health Policy and Strategic Plan was a frequently mentioned outcome:

One way the Mental Health Coalition has been able to impact the country is through the development of the policy which … has impacted the country [by] getting the political will to operate or develop programs and also even educating people, people to have a different view about mental health in the country. (KI, NGO representative)

Respondents also mentioned the MHC's work with the government on the inclusion of mental health issues in the poverty reduction strategy paper, the Agenda for Prosperity (33). However, differences in opinion emerged as to the quality of the results. One KI, a government official, described the MHC working together with the government as a ‘huge success’ as they ‘were able in the end to have mental health as a programme within the Ministry of Health, which has never been done before’. Conversely, another KI, a development partner, explained, ‘I don't think anybody was 100% happy with what came out, but something is better than nothing, I suppose’.

**Table 5:** Key achievements of the Mental Health Coalition – Sierra Leone

• **National Mental Health Policy**: Founding members of the MHC were involved with the initial drafting and validation of the National Mental Health Policy (31) in 2009. The MHC when formed in 2011 took on the issue of the Policy as a key target, resulting in the identification of the need for a formal launching as the integral stumbling block for political acceptance of the Policy and working to promote its eventual launch in 2012.
• **Mental Health Strategic Plan**: The MHC reinforced the MoHS’ coordination of the drafting and circulation of the Mental Health Strategic Plan (32) which was presenting at the launching of the Policy.
• **Agenda for Prosperity**: The MHC supported the Office of the President's Strategy and Technical Unit in its process of drafting of Sierra Leone's second Poverty Reduction Strategy Paper (PRSP), *the Agenda for Prosperity* (33). The MHC viewed its success in the inclusion of key elements of the Mental Health Policy in the drafts of the PRSP II, with the outcome of mental health mentioned in the final draft. The MHC supported the establishment of the MoHS’ Mental Health Steering Committee, for which its members holds key supporting roles.
• **Annual Mental Health Conference**: The MHC initiated an annual conference on mental health in Sierra Leone, bringing together both national stakeholders and international participants****.

Respondents mentioned achieved aims in terms of its annual conference and advocating for the rights and supporting basic needs of service users. A KI, an NGO representative, explained an instance of the MHC mediating in a dispute regarding worker compensation at the psychiatric hospital:

When the workers went on strike, and then they locked the patients in, and they refused access to carers … the Coalition was able to … get them to talk to us …. We had discussions [with Labour Congress and MoHS]. And at the end of the day, the workers had rights, and their requests were met, but in the process we made them to know that you cannot use the client as your weapon to fight your battle. So that is one action that I am proud of that the Coalition took. But also in the process, we were able to get them to agree that we could at least come with a day's meal to the inpatients at [Sierra Leone Psychiatric Hospital].

### Enabling factors

The analysis uncovered enabling factors for advocating for mental health in Sierra Leone. Strategies for networking emerged as enabling factors, in particular establishing links with key partners and stakeholders in mental health nationally, regionally, and globally (see Table 3, networking). Establishing connections with government goes beyond formal ties, in particular with strategic entry points, as KI, a NGO representative, described:

The right entry point … if we had started off … trying to storm the Office of the President, I believe we would not have made this progress. But we started off with the person closest to us … it's not only an official relationship … there is that comradeship, there is a friendship … from him now, we slowly are infiltrating into the government departments.

Respondents emphasised the MHC's interaction with the MoHS as an enabling factor. In particular, ensuring the active involvement of MoHS, working towards the same goal, and continually and gently pushing the government and supporting their work until they fully appropriate objectives, were mentioned:

When at the Steering Committee Meeting, I sense that Government … heavily relies on the activity of the Coalition. So we've made ourselves a reliable group to push mental health issues in the country. (KI, NGO representative)

Participants underscored several communication strategies (see Table 3, awareness raising) as enabling awareness raising. Respondents highlighted MHC members acting as peer advocates, championing mental health in their own communities and networks, as an enabling factor for awareness raising. Respondents described strategies in awareness raising as not necessarily equating to effective change, but as ‘setting the stage’ for possible future change. One KI, a NGO representative outlined:

The fact that they were executed by the Coalition is the first thing. Whether something begins to happen in the physical … entity or not, we know that a message has gone down mentally. So, at one time or the other it would surface, and those who are supposed to take action would take it … a seed has been sown. Whether the seed will germinate or die, we are yet to prove over the years.

### Barriers for advocacy aims

Respondents pointed to barriers to mental health advocacy that were evident across several themes. Some of these barriers were previously identified as enabling factors, for example, working alongside government. Respondents also pointed to the identification of mental health as an issue and ‘getting everyone on board’ to support mental health, from the government to the community level, as barriers. Respondents flagged awareness raising with Mental Health Nurses and MHC members underlining that generally more sensitization is needed. Stigma, including at the level of decision makers, was highlighted as a barrier specific to awareness raising.

Despite respondents pointing to small, favourable shifts in political will and mindset change, government leadership, including in planning, remains a challenge. Subsequently, the implementation of the policy ‘might be one big problem’ (KI, NGO representative). The lack of a holistic view of mental health and associated services was also a related barrier, with one KI asking: ‘How do you integrate mental health … so people begin to see health as wholeness of mind and body?’

In regards to networking, respondents outlined the small numbers of mental health partners as a barrier. The lack of public awareness of the MHC activities was pointed out as a barrier, with MHC members themselves expressing their own lack of knowledge. A KI, a government official, described traditional healers as not comfortable as they did not feel included and stand to lose financially with scaling up of mental health services, despite others mentioning that this group is well-represented in the organisation.

Respondents declared resource mobilization for both services and for advocacy activities as a barrier, with a compound effect of mental health as the lowest health priority amongst competing development concerns for both the government and donors.

## Discussion

The results of this study provide insights into the perspectives of mental health stakeholders in Sierra Leone on the impact of a local mental health advocacy organisation. It unpacks some of the dimensions of enabling factors and barriers to its work.

### Scaling up mental health care

Study respondents listed several of the MHC's advocacy aims since the organisation was founded in 2011, including those achieved and those that are the ongoing focus for the MHC's activities. The identified aims are in line with the growing body of evidence (34, 35) and the priorities of the Global Mental Health Movement (14, 36), which call for a scaling up of mental health care in order to improve the lives of people with psychosocial disabilities in low- and middle-income countries. Simultaneously, evidence indicates that it is advocacy groups that are best poised for pushing governments to make positive changes in their country's national mental health plans (14, 23). By utilizing arguments based in evidence, the MHC further strengthens the messages used in advocacy campaigns (37).

### Policy development

The results of this study point to slow, but positive change (23), despite the low priority of mental health in the policy sphere in Sierra Leone and globally (21). To apply two pillars of a framework (38) for global priority of health issues to a national stage, the alignment of actor power – the strength of those concerned with the issue, with the political context, enabled the political acceptance of the National Mental Health Policy and Strategic Plan. Both documents were drafted and a validation meeting held in 2009 (39), but did not gain acceptability in the political sphere until the MHC mobilized civil society and the policy community, including the Mental Health Steering Committee and the First Lady of Sierra Leone to publically and formally launch the policy documents on World Mental Health Day 2012 (39). Such flexibility towards the pragmatic use of opportunities as they arise was highlighted by a respondent in this study as an enabling factor, mirroring recommendations for low-resource settings in the scale up of mental health services (14).

### Implementation of policy guidelines

Despite the success of supporting the launch of the National Mental Health Policy, stakeholders’ recommendations revolved around implementation of the policy in the realm of service provision. This mirrors findings from Liberia (14), Pakistan, and South Africa (12) that the success of policy reform at the national level does not necessarily translate into implementation.

### Self-representation

Representation of service users in the organisation and their viewpoints in its advocacy work was not evident in the analysis. Membership includes service user representation, but in a context of stigma, the MHC does not actively encourage disclosure of disability status.

### Resource challenges

Financing and budget allocation for mental health remain an obstacle in Sierra Leone, confirming similar findings in low-resources settings of the impact of limited resources and competition for resources (40). The Freetown City Council budget provides the only support for the psychiatric hospital, whereas district services are dependent on District Health Management Teams electing to finance such services from the Basic Package for Essential Health Services district budget. Moreover, in a health care system with significant resource support from donors, their lack of support often equates to lack of available resources. However, renewed emphasis on the importance of psychosocial support by the Government of Sierra Leone (GoSL) in the wake of the Ebola crisis is initiating a change into recognition of need and generation resources for such care (41).

### Legislative reform

This study highlights the priority of legislative reform for mental health stakeholders in Sierra Leone. The Lunacy Act of 1902 (42), a provision passed down by the former colonial government that does not recognise the rights of persons with psychosocial disabilities and contributes to their discrimination and alienation from society (21, 24), is in need of revision, along with other legalisation that impacts on the rights of mentally ill. Male family members of service users emphasised challenges regarding the role of stigma in accessing housing and the fact that ‘en government nor get da authority day; E kin say are nor want di porsin na mi ose, government nor go say pao pa’.^1^ Respondents pointed to the fact that the MHC has formed a subcommittee on this topic; however, no successful outcomes from its efforts were reported, highlighting the need for further, sustained action towards this aim in the reality that, as a male MHC member pointed out: ‘In orda for change law na Salone, di process, e too long, en almost yu kin see if yu nor kin get di political will of di politicians, especially wae na an issue wae nor dae bring moni for dem, so dem nor dae improve’.^2^

### Relationship with government

A significant enabling factor is the group's approach to interacting with the MoHS, with strategic engagement and effective working relationships developed with government and stakeholders (43). As well as having a political mandate, national governments ‘have to be in the driver's seat for creating coordination mechanisms that harmonize efforts of different partners and agencies’ (44). The MHC approach follows this recommendation by allowing for the MoHS to take the lead through the coordination mechanism that the Mental Health Steering Committee provides, while providing technical support and execution of tasks under their leadership.

### Networking for advocacy

The study supports literature that outlines a need for coordinated advocacy movements for mental health (45) to spur demand for change in the context of low political will of public policymakers (22, 46). Some resources now exist to support this (47, 48). In addition, the findings support the positive outcomes of strengthening relationships with regional mental health stakeholders (23).

## Conclusions

The study uses the experience of a stakeholder coalition in Sierra Leone to explore mechanisms for successful advocacy for mental health in a low-resource setting. Key enabling factors to overcome barriers of low prioritisation of mental health, low political will, and poor investment, were: the use of networks with consistent messages; the importance of relationships with advocacy targets, and giving them a sense of ownership; and using effective tools opportunistically for awareness raising.

In addition to the moral argument for including stakeholders in policy decisions that affect them, this group's contributions to milestones in scaling up mental health add to the growing evidence that stakeholder groups are effective partners in strengthening mental health systems in low-resource settings.

## Footnotes

1 And government lacks that authority; if the landlord rejects the individual from living in his house, government cannot insist that he must stay.

2 To change laws in Sierra Leone, the process is too long. And you can see if you don't have the political will of the politicians, especially so if it is an issue that does not bring money for them, they do not improve.
